# Causal Role of the Right Dorsolateral Prefrontal Cortex in Organizational Fairness Perception: Evidence From a Transcranial Direct Current Stimulation Study

**DOI:** 10.3389/fnbeh.2020.00134

**Published:** 2020-08-28

**Authors:** Xi Li, Guanxing Xiong, Zhiqiang Dong, Shenggang Cai, Jun Zhao, Zhe She, Yuchen Guo

**Affiliations:** ^1^Key Lab for Behavioral Economic Science and Technology, South China Normal University, Guangzhou, China; ^2^School of Economics and Management, South China Normal University, Guangzhou, China

**Keywords:** right dorsolateral prefrontal cortex, organizational fairness perception, modified ultimatum game, transcranial direct current stimulation, emotional control

## Abstract

The right dorsolateral prefrontal cortex (rDLPFC) plays an essential role in social decision-making. Although several neural imaging studies have provided evidence that the rDLPFC is correlated with fairness perception, little research has investigated the causal effect of this encephalic region on individuals’ consciousness, particularly perceptions of organizational fairness. The present study explores the causal relationship between the rDLPFC and organizational fairness perception by using brain modulation techniques. Healthy participants received transcranial direct current stimulation (tDCS) and fulfilled the modified ultimatum game (UG) in the sham-controlled experiment. Our results showed that only cathodal stimulation of the rDLPFC resulted in increasing rejection offers compared with the sham stimulation in conditions of disadvantageous inequity. No differences were found between the anodal and sham stimulation in any inequity condition. This study strengthens the main functional effects of the rDLPFC in negative emotional control in relation to organizational fairness perceptions.

## Introduction

Organizations, and particularly the social relationships they involve, are based on fairness values. Organizational fairness perception, that is, the perception of equity in the distribution of resources or outcomes (McFarlan and Sweeney, 1992), is a strongly motivating driving force for the adjustment of organizational behavior to maximize interests. The evaluation of various perspectives is essential and differs from the evaluation of organizations’ roles in fairness perception. Numerous scholars in the management domain have focused on the investigation of perceived fairness regarding reward distribution among group members, viewing organizations as a background or context. Questionnaire methods have typically been applied to identify organizational unfairness perception leading to negative outcomes such as theft, dissatisfaction, and poor performance (Greenberg, 1990; Colquitt et al., 2001). However, both organizations and individuals seek to gain benefits and avoid losses. In other words, organizations should also be considered a decision-making body or behavioral subject pursuing the maximization of its own interests. Thus, fairness perception can be discussed from the perspective of games between an organization and its members.

Research has explored neural substrates of fairness perception using the ultimatum game (UG) paradigm, in which the participants decide whether to accept a division of money suggested by a proposer (Sanfey et al., 2003). The UG is a widely used laboratory tool for investigating economic decision-making, and it is an acknowledged method for examining perceived fairness or fairness preferences. Güroğlu et al. (2010) argued that neural effects in the UG were independent of context, which suggested that regulation and control are involved in fairness perceptions generally. However, studies on neural networks involved in perceived fairness in the UG have mostly focused on reciprocal fairness at the interpersonal level by using an anonymous self-centered task (Civai et al., 2015), and fairness perception at the organizational level has been ignored.

In the traditional UG (Güth et al., 1982), an individual as a proposer offers a division of a sum of money to another individual as a responder, who decides to accept or reject this offer. If the responder accepts the division, both receive the suggested amounts. If the responder rejects the division, neither receives anything. The position of these two parties is unequal. The proposer is in the dominant position, but the responder has the “voice” to judge the results of distribution. The right to a “voice” is considered the factor majorly affecting the perceived fairness of distribution (van Dijke et al., 2018). It is extremely similar at the organizational level. No matter what type of organization is involved, the organization itself is viewed as the allocator of outcomes and resembles the “proposer.” In other words, powerful and authoritative organizations assume the dominant position without exception; individuals are in a disadvantaged and subordinate position as “responders.” In reality, to achieve legitimacy and democracy, individuals are encouraged to express their opinions in modern organizations. With the rights of participation, members are even willing to reject potential benefits to present their dissatisfaction regarding results. Moreover, regarding classifications of unfair offers in the traditional UG, 9:1 or 8:2 splits were viewed as extremely unfair offers, 7:3 splits were viewed as moderately unfair, and 5:5 splits and even 6:4 splits were viewed as fair (Yamagishi et al., 2012). In general, individual proposers had no incentive to offer others >50% of the total money. However, the situation is completely different when an organization acts as the proposer. An organization is a social unit of people with a particular purpose, which may offer a member a greater proportion of benefits to emphasize long-term sustainability. Thus, advantageous inequity (responders receive more than proposers) and disadvantageous inequity (responders receive less than proposers) were adopted in our research instead of fair and unfair offers to explore organizational fairness.

The neural region of the brain explored in this study was the right dorsolateral prefrontal cortex (rDLPFC), which is thought to play a major role in the perception of distributive fairness (Sanfey et al., 2003). It is strongly associated with self-control (Hare et al., 2009), response selection (Hadland et al., 2001), and motor planning and target maintenance (Barbey et al., 2013; Colombo et al., 2016). By applying the UG paradigm, Knoch et al. (2006) found that low-frequency repetitive transcranial magnetic stimulation (rTMS) on the rDLPFC reduced subjects’ willingness to reject their partners’ intentionally unfair offers, with individuals who received this stimulation less able to punish others’ unfair behaviors and more able to rationally consider their own self-interest. Other evidence, combining fMRI and transcranial magnetic stimulation (TMS), showed that the rDLPFC (but not the left DLPFC) and the posterior ventromedial prefrontal cortex (pVMPFC), as well as their connectivity, contributed to the evaluation of unfair offers and to the subsequent costly decision to reject them (Baumgartner et al., 2011). Notably, the findings from the UG experiments indicated that responders’ reciprocal fairness perceptions were affected by not only the outcomes of distribution itself but also the underlying intention of proposers (Güroğlu et al., 2011). In a study by Falk and Fischbacher (2006), some second movers punished unfair offers and rewarded advantageous offers, even if offers were randomly determined. Taken together, these results of fairness perception involve only the individual level; to our knowledge, no study has assessed causal links to the organizational level.

This study was aimed to provide neural evidence through the identification of a causal relationship between rDLPFC function and organizational fairness perception. We applied transcranial direct current stimulation (tDCS) to induce changes in the activity of the rDLPFC compared with the sham stimulation, with the final goal of altering subjects’ performance during the modified UG experiment. Because another study investigated the effects of cathodal tDCS applied over the rDLPFC on interpersonal UG behavior (Knoch et al., 2008), we attempted to maintain the stimulation at a consistent level. We hypothesized that, compared with the sham condition, the cathodal stimulation of the rDLPFC significantly increases rejections of unfair offers when an organization acted as the proposer. Furthermore, anodal tDCS stimulation enhances cortical excitability, whereas cathodal stimulation reduces cortical excitability (Nitsche and Paulus, 2000). Anodal and cathodal simulation both modulate brain activity, resulting in opposite effects. Considering the integrity of the experimental design, we also hypothesized that, compared with the sham condition, the stimulation of rDLPFC through anodal tDCS significantly reduces the numbers of rejections of unfair offers when an organization served as the proposer. A 3 × 2 mixed-model analysis of variance (ANOVA) for the factors of treatment (cathodal, anodal, and sham), inequity type (advantageous and disadvantageous inequity), and treatment × inequity type interaction was adopted to examine these two hypotheses.

## Materials and Methods

### Experimental Design

According to literature on tDCS, two lines of tDCS stimulation design have been used. Scholars used within-subject comparison to apply all three stimulation conditions (anodal, cathodal, and sham) for each participant (Hecht et al., 2013). To avoid the carryover effects of prior stimulation sessions, tDCS simulations lasted a minimum of 2 days (~47 h) apart. Between-subject design has also been applied for tDCS stimulation, such as in Oldrati et al. (2016) and Gaynor and Chua (2017). Different participants received different tDCS stimulations randomly. Although the first method is better for explaining causal effects, experimental subjects make the same behavioral decisions three times and thus their answers may be affected. The recruitment of subjects who are willing to receive three rounds of brain stimulation is also difficult. Because more credible results can be obtained by increasing the numbers of subjects, three rDLPFC stimulation types were used for subjects in our research. Moreover, the study was designed as a single-blinded, sham-controlled, and mixed experiment. For the sham stimulation, the procedures were the same, but the stimulator was activated only for the initial 30 s. This was designed to ensure that participants were effectively blinded (Sellaro et al., 2016). In the interview after the experiment, all participants in this group had felt a tingling sensation associated with tDCS and were unaware that the treatment had faded. In addition, for the measurement of organizational fairness perception, within-subject comparison was employed, which was divided into advantageous inequity (receiving more) and disadvantageous inequity (receiving less) as per Gao et al. (2018).

### Participants

Ninety right-handed healthy volunteers were recruited. They were randomly assigned to brain stimulation groups (30 for anodal stimulation, 30 for cathodal stimulation, and 30 for sham stimulation). All participants had normal or corrected-to-normal vision and were naïve to tDCS and UG tasks. None of them had a serious medical condition or a history of neurological diseases or psychiatric disorders. Participants received basic compensation of ¥50 (approximately US$7.2) for their attendance and were informed that they could obtain more money during the UG depending on their choices. Each participant was paid an additional ¥15 (approximately US$2.14) for the UG on average. The total compensation that one participant finally received was approximately equivalent to five times the minimum hourly wage in Guangzhou. The whole experiment lasted for approximately 50 min. Informed written consent was obtained from each participant before the experiment. The study was approved by the Institutional Ethics Committee of South China Normal University. Safety procedures were followed in accordance with non-invasive brain stimulation (NIBS) indications (Poreisz et al., 2007).

### Stimulation Parameters

In the past two decades, >1,000 articles have been published in which tDCS tools are used (e.g., Jantz et al., 2016; Xiong et al., 2019). A simple, painless, and noninvasive technique is used for the modulation of brain activity, with a low-intensity direct current applied. This is adjusted to induce cortical excitability in the target area without any physiological damage to the participants. Anodal stimulation may facilitate behavioral effects, whereas cathodal stimulation may inhibit them (Stagg and Nitsche, 2011). We used tDCS to investigate the causal effects of human brain functioning on organizational fairness perceptions. Compared with other methods of neurostimulation, tDCS provides the benefits of more easily allowing placebo-controlled studies to use sham stimulation (Gandiga et al., 2006). Current was delivered through a battery-driven constant stimulation (NeuroConn DC-STIMULATOR, Germany) using two saline-soaked surface sponge electrodes (5 × 7 cm^2^). For stimulation of the rDLPFC, the anodal or cathodal electrode was placed over F4 (according to the international EEG 10/20 system) and the reference electrode over the other side of the deltoid muscle, which is a recommended and commonly used reference site (Priori et al., 2008). The current was constantly maintained at 1.5-mA intensity with 30 s of ramping up and down. The participants were asked to complete the experimental task and questionnaire after 20 min of stimulation. To avoid disturbance, all participants wore the tDCS devices until they finished the remaining experiment (approximately 20 min). All stimulation parameters complied with safety guidelines. The average impedance achieved and maintained during tDCS was 5.3 kΩ.

### Mortified UG Task

UG is the most widely used decision-making task for the study of individual responses to fairness perception, including several neuroscience studies (Boksem and Cremer, 2010; Civai et al., 2015; Zheng et al., 2015; Blair-West et al., 2018). In the game (Güth et al., 1982), one player (i.e., proposer) distributes available wealth; the second player (i.e., responder) can choose to either accept or reject this offer. If an offer is accepted, then funds are allocated per the proposal. If it is rejected, neither party receives any money. However, one major theoretical limitation of these traditional games is that the results are suitable for explaining only anonymous interpersonal justice rather than perceived organizational fairness. Whether the same behavioral and cognitive mechanisms are applicable if an organization acts as a proposer merits consideration. To achieve this objective, we were motivated to develop a modified version of the UG with a specific organizational context.

OTree (Chen et al., 2016) was used to present participants with a scenario from Zhang and Zhou (2018): “Imagine that your college has received a donation of ¥150,000 (approximately US$2,143), and 10% is dedicated to student welfare. The donors specify that, if one student rejects the money, the corresponding donation will be deducted (in a similar manner to the punishment of proposers in the traditional UG), and the allocation plan should also be consulted for each student’s opinion. This college has 500 students, and every student obtains ¥30 (150,000 × 10% ÷ 500 = 30) according to the corresponding distribution ratio. However, the remaining donation is certainly not to be used for student welfare, such as by improving college facilities or faculty welfare.” This was in fact a cover story and helped ensure that the context was the same for every subject. For the manipulation of inequity type, the aforementioned story appeared on the screen in every decision. In other words, the main body of the scenario was the same, but the distribution proportion changed randomly. We viewed 10, 20, 30, and 40% as disadvantageous inequity and 60, 70, 80, and 90% as advantageous inequity. In addition, because monetary incentives are required to create as real an environment as possible, tokens were used in the scenario description that could be exchanged for real money at a certain exchange ratio (10%). All participants were required to serve as responders and selected acceptance or rejection for each trial by clicking the mouse. They made only their own decisions and had no idea about the choices of others. The mean number of rejections of modified UG offers in various blocks were compared after the tDCS stimulation.

### Procedure

**Figure 1:**
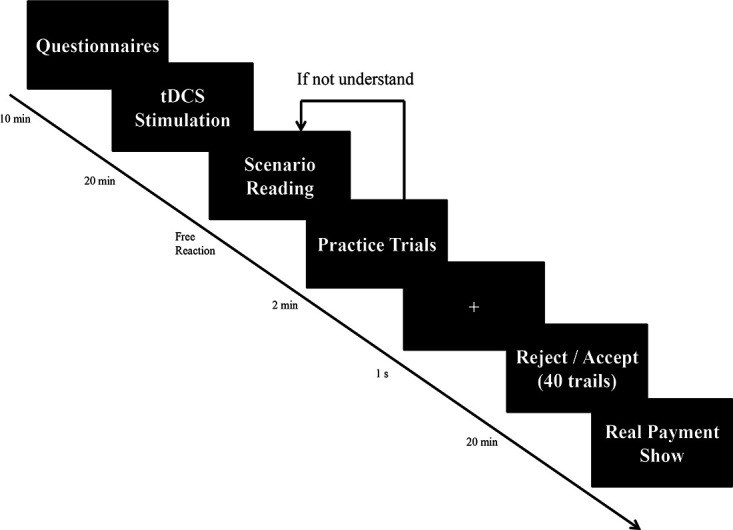
Schematic representation of the experiment procedure.

Participants were informed of the nature of the experiment, particularly the tDCS methodology and additional payment for the UG task. On arrival at the laboratory, experimental participants were asked to provide the written materials and underwent preparation. Participants then randomly received either active or sham tDCS to the rDLPFC for 20 min. During this period, they were not permitted to perform any activities. Any discomfort could be announced. The participants then read the task instruction on the OTree screen and completed three practice trials. If the subjects passed the test, they participated in the formal experiment. If not, they reread the task instructions until they were able to answer the test questions correctly (Figure 1). We used practice trials to ensure that all participants understood the scenario and made selections freely.

There were five sessions in the formal experiment. Each session contained eight trials, the distribution proportion of which was from 10 to 90% (50% not included). They were randomly displayed on the screen by Otree software. For avoiding the sense of the task being boring and repetitive, there were very small changes about the donation amount in each session, such as 150,000, 150,100, to 150,400. The sequence was also randomly presented for different participants. We assumed that these small changes were negligible and all the sessions could be viewed as equal. After the completion of all experimental assignments, we randomly selected an option, according to participants’ responses, of providing an additional payoff in the UG game. In the aforementioned example scenario, if the chosen offer was accepted when the college allocated 10% of the donation to students, the participant obtained an additional ¥3 (30 tokens), whereas if the offer was rejected, they received nothing except for basic attendance compensation. The payment was finally shown on the screen to ensure the reliability of subjects’ decisions. After the experimental sessions, all participants completed an interview to evaluate whether the stimulation protocol affected the sensations experienced that could potentially influence subject performance, particularly for the sham group. No such effects were reported.

### Statistical Analysis Methodology

To enable replication, the details of statistical analysis methodology are described in this section. In the original dataset, we obtained forty decision results (accept = 0; reject = 1) per subject. The total number of rejections (rejection size) was then calculated for each block (advantageous and disadvantageous inequity). A 3 × 2 mixed-design ANOVA was used to analyze the effects of the between-subjects factor (anodal, cathodal, and sham), within-subjects factor (inequity type), and treatment × inequity type interaction. Before data testing, unusually large or small outliers were examined with reference to Dewasurendra et al. (2017). Moreover, *post hoc* analysis was applied to compare the mean number of rejections between treatment conditions. Because Croson and Gneezy (2009) argued that gender differences might exist in the UG, we also examined gender as the covariable in the ANOVA.

## Results

**Figure 2:**
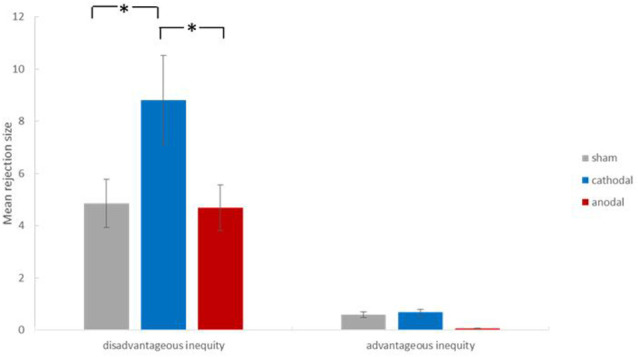
Results of 3 (treatment) × 2 (inequity type) mixed-model analysis of variance (ANOVA) analysis. The bar plots showed the mean number of rejection offers. Under the condition of disadvantageous inequity, the mean of rejection size in the cathodal group was significantly higher than that in the sham group (*p* = 0.03), and higher than that in the anodal group (*p* = 0.02). Error bars indicate s.e.m. *p < 0.05.

**Figure 3:**
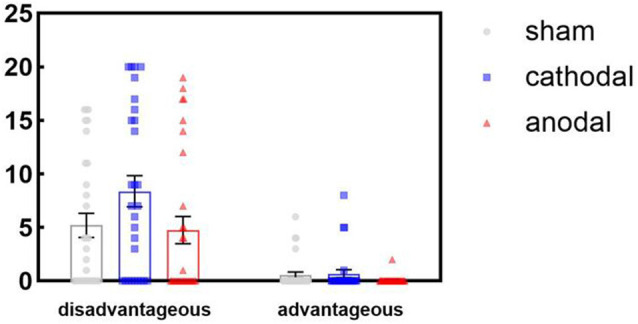
Results of 3 (treatment) × 2 (inequity type) mixed-model ANOVA analysis. The dot plots presented the total number of rejection offers of some participants.

SPSS (version 21) was used in our study. The data elimination standard of excluding values outside *Mean* ± 3 *SD* was used to omit three sample data from the overall analysis. Therefore, our valid sample size was 87 (30 in the anodal group, 28 in the cathodal group, and 29 in the sham group), comprising 42 men and 45 women, with an age range of 18–24 years (mean = 20.38, standard deviation = 1.21). We performed a two-way ANOVA with the treatment (anodal, cathodal, and sham stimulation) as a between-subjects factor and inequity type (advantageous and disadvantageous inequity) as a within-subjects factor. The 3 (treatment) × 2 (inequity type) mixed-model ANOVA revealed the following: the main effect for treatment was significant (*F*_[2,84]_ = 3.24, *p* = 0.04, *η*^2^ = 0.07); least significant difference (LSD) *post hoc* analysis showed that the mean of the rejection size in the cathodal group (*M* = 4.75) was significantly higher than that in the sham group (*M* = 2.72) and anodal group (*M* = 2.38). The main effect for inequity type was significant (*F*_[1,84]_ = 70.46, *p* < 0.001, *η*^2^ = 0.46); LSD *post hoc* analysis showed that the mean number of rejections for disadvantageous inequity (*M* = 6.13) was higher than that for advantageous inequity (*M* = 0.44). In addition, the interaction of the treatment by inequity type was significant (*F*_[2,84]_ = 3.26, *p* = 0.04, *η*^2^ = 0.07). The descriptive statistics can be seen in Table 1. Simple effect analysis showed that, subject to the condition of disadvantageous inequity, the mean number of rejections in the cathodal group was significantly higher than in the sham group (*p* = 0.03) and higher than in the anodal group (*p* = 0.02; Figures 2, 3). No significant differences existed between the anodal and sham group (*p* = 0.93). For the condition of advantageous inequity, no significant differences were found among the anodal, cathodal, and sham group (*F*_(2,84)_ = 1.55, *p* = 0.22). Furthermore, to avoid the interference of gender difference, we added gender as the covariable in our 3 × 2 ANOVA analysis. The main results were unaffected by gender. In addition, the effect of task type × gender (as a within-subjects factor; *p* = 0.73) and gender as a between-subjects factor (*p* = 0.99) were both nonsignificant.

**Table 1:** Descriptive statistics of each group (*M* ± *SE*).

Stimulation type	Disadvantageous inequity	Advantageous inequity
Anodal	4.70 ± 0.87	0.07 ± 0.01
Cathodal	8.82 ± 1.70	0.68 ± 0.13
Sham	4.86 ± 0.92	0.59 ± 0.11

## Discussion

The tDCS technique yielded evidence regarding the organizational fairness perceived by UG responders of using stimulation applied over the rDLPFC. Notably, unlike most studies discussing altruistic helping behaviors or reciprocal fairness perceptions in an interpersonal context (Nihonsugi et al., 2015; Hu et al., 2017), we focused on the organization’s role as the proposer in a modified UG task. Our results showed that the neural effects of the rDLPFC on organizational fairness perception were dependent on inequity type. Subject to the condition of disadvantageous inequity, cathodal tDCS significantly increased the mean number of rejections in the UG compared with the sham stimulation. In other words, when receiving less than the organization (proposer), individuals tended to refuse more offers by cathodal stimulation. Subject to the condition of advantageous inequity, however, no effects were observed. Furthermore, anodal tDCS activation triggered no causal effects subject to any types of inequity.

First, the effects of tDCS stimulation shall be discussed. Miniussi et al. (2013) questioned whether tDCS stimulation was directly applicable in the cognitive neuroscience field because the final behaviors were highly complicated. So we aimed to distinguish this. Determining whether a deviation from normal behavior consists of impaired response or improved performance is difficult (Civai et al., 2015). However, if the normal baseline response of the sham group is definitively obtained, a deviation from the norm can be found. Reasonable deductions by theories also provide some helpful explanations. In the UG task, optimal decision-making by responders was considered an acceptance of all offers to increase self-interest, whereas any rejections were considered negative emotional reactions (Charness and Rabin, 2005). When an authority on behalf of a legal organization acted as the proposer in our study, cathodal tDCS stimulation nonetheless resulted in significantly greater mean numbers of rejections compared with the baseline response. Thus, our results contribute to providing new proof for a causal link between rDLPFC and fairness perception. However, tDCS is a diffuse form of noninvasive brain stimulation; therefore, the possibility that other brain regions or networks are involved is difficult to eliminate. More focused forms of brain stimulation, such as TMS or high-definition tDCS, might be required for more detailed answers.

In neural science, two distinct explanations currently exist for the effects of rDLPFC function on fairness perception in the UG. One is the emotional control hypothesis (e.g., Sanfey et al., 2003; Civai et al., 2010). This research features the argument that in order to maximize self-benefit, individuals must control the emotional refusal tendency associated with unfair offers to punish the proposers and control the negative emotions caused by unfair distribution outcomes. The alternative explanation is the cognition enhancement hypothesis (e.g., Knoch et al., 2006; Baumgartner et al., 2011). These researchers have emphasized that the rDLPFC is responsible for self-regulation, goal maintenance, and manipulation of information in working memory and it therefore has an extremely prominent role in rational behaviors. Humans attempt to limit the effects of self-interest through the development and enforcement of social norms to achieve long-term goals. Both arguments are consistent with dual-system approaches that indicate the fundamental differences between emotional (impulsive) and rational (cautious) systems or between automatic and controlled processes (McConnell and Rydell, 2014). However, despite copious research, the functional aspect of the rDLPFC that causes the rejection of unfair offers remains unclear (Speitel et al., 2019). Here, we attempted to answer this question through differentiation of the inequity type. We assumed that the subjects’ experiences of disadvantageous inequity were significantly related to negative emotional arousal elicited by the receipt of smaller rewards compared with proposers (Gao et al., 2018). For advantageous inequity, when receiving more than proposers, subjects may not consider only short-term benefits for themselves, but they may demonstrate advanced social cognition, such as through recognition of the norm and corresponding adjustment to generate long-term effects (McAuliffe et al., 2017). Our scenario in the experiment was an example in which contributions other than direct payments might have indirectly benefitted the students participating, such as through improvement of college facilities or through increasing faculty welfare. If the subjects’ decisions were from a long-term and macroscopic perspective, they might even have rejected the offers advantageous to them. After the experiment, we interviewed some participants who refused advantageous offers. One woman said that she might have spent the money extravagantly and would ultimately derive more benefit if the college retained more of the donation. We believed that people acted in opposition to advantageous inequity toward organizations, particularly in collectivist cultures, such as in China.

The current data demonstrated that rDLPFC inhibition was related to more rejections subject to a disadvantageous inequity condition, and this finding was consistent with those of other studies (Grecucci et al., 2013; Morewedge et al., 2014). This result suggests that the rDLPFC controls negative emotional reactions to perceived unfairness, even when the organization acts as a resource allocator. Thus, the emotional regulation by the rDLPFC, which has a part in self-control functions, is highlighted. For advantageous inequity UG offers, no differences were found between the group for anodal or cathodal tDCS and the sham stimulation group. In our study, we cannot claim that the rDLPFC processes the integration of fairness norms in the long run to provide immediate benefits. Future studies should use more experiments to provide further evidence on this rDLPFC function.

The present study has some limitations. First, individual differences must be represented for the social brain to be understood. Clearly, there is a growing recognition that personality traits can help explain the heterogeneous responding within many economic games (Zhao and Smillie, 2015). For example, Yamagishi et al. (2012) found that the personality trait of assertiveness, in contrast to prosocial behavior, predicted the rejection rate of unfair offers in the UG. Second, in additional studies, expanded sample sizes and within-subject designs should be considered. Consistent with Oldrati et al. (2016) and Gaynor and Chua (2017), we could not use a within-subjects design, which would have meant presenting participants with the same set of decision-making questions three times. Future studies may benefit from mixed experimental designs in an operable and rigorous paradigm. Finally, answering the call of Nihonsugi et al. (2015) to explore the neural mechanism for intention-based economic decisions in broader social contexts, we designed an experiment to identify the causal effects of the rDLPFC on perceived organizational fairness using the UG paradigm. Although Güroğlu et al. (2010) argued that the DLPFC’s role of regulation and control in fairness perceptions was independent of context, multiple contexts provided in the experiment would improve reliability. Further relevant research should refer to studies such as Luo et al. (2017) to consider various organizational contexts or backgrounds.

## Conclusion

The present study applied tDCS to modulate the rDLPFC to alter social decision-making in relation to organizational fairness perception. To generate the various functions of rDLPFC, we created a modified UG task to highlight advantageous and disadvantageous inequity conditions. Our results indicate that, subject to the condition of disadvantageous inequity, cathodal tDCS significantly increases the mean number of rejections for UG offers compared with the sham stimulation; the same effect is not observed for advantageous inequity conditions. No differences are found between the group for anodal tDCS and the sham stimulation group in any conditions of inequity. We further inferred that the rDLPFC has a role in the self-regulatory system involved in people’s reactions to offers characterized by disadvantageous inequity when an organization serves as a proposer. The rDLPFC is also suggested to be more likely to be responsible for emotional control when perceiving organizational unfairness.

## Data Availability Statement

The datasets generated for this study are available on request to the corresponding author.

## Ethics Statement

The studies involving human participants were reviewed and approved by the institutional ethics committee of South China Normal University. The participants provided their written informed consent to participate in this study.

## Author Contributions

XL, GX, ZD, and SC designed experiment. XL, JZ, ZS, and YG performed experiment. GX analyzed the data. XL and GX drew figures, wrote the manuscript and revised the manuscript. All authors contributed to the article and approved the submitted version.

## Conflict of Interest

The authors declare that the research was conducted in the absence of any commercial or financial relationships that could be construed as a potential conflict of interest.
